# Mathematical Analysis of a Mathematical Model of Chemovirotherapy: Effect of Drug Infusion Method

**DOI:** 10.1155/2019/7576591

**Published:** 2019-03-11

**Authors:** Joseph Malinzi

**Affiliations:** Department of Mathematics, University of Eswatini, Private Bag 4, Kwaluseni, Eswatini

## Abstract

A mathematical model for the treatment of cancer using chemovirotherapy is developed with the aim of determining the efficacy of three drug infusion methods: constant, single bolus, and periodic treatments. The model is in the form of ODEs and is further extended into DDEs to account for delays as a result of the infection of tumor cells by the virus and chemotherapeutic drug responses. Analysis of the model is carried out for each of the three drug infusion methods. Analytic solutions are determined where possible and stability analysis of both steady state solutions for the ODEs and DDEs is presented. The results indicate that constant and periodic drug infusion methods are more efficient compared to a single bolus injection. Numerical simulations show that with a large virus burst size, irrespective of the drug infusion method, chemovirotherapy is highly effective compared to either treatments. The simulations further show that both delays increase the period within which a tumor can be cleared from body tissue.

## 1. Introduction

Tumors possess mechanisms that suppress antitumor activity such as ligands that block natural killer cells and cytotoxic tumor infiltrating cell functions [1]. Greatly because of this, successful cancer treatment often requires a combination of treatment regimens.

Nearly all traditional monotherapies, including chemotherapy, surgery, and radiation therapy are not a definite cure for cancer and are highly toxic [2]. Chemotherapy for example, which is the most commonly used regimen, involves the use of medical drugs to lyse cancer cells. These chemotherapeutic drugs circulate in the body and kill rapidly multiplying cells nonselectively, which ultimately results into the destruction of both healthy and cancerous cells [2, 3]. Chemotherapy can thus be toxic to a patient with adverse side effects and can also damage their immune system [2].

Presently, combination cancer treatment is a centerpiece of cancer therapy [4]. The amalgamation of anticancer drugs increases efficacy compared to single-drug treatments. Further, anticancer drug combination provides therapeutic benefits such as reducing tumor growth, arresting mitotically active cells, reducing the population of cancer stem cells, and inducing apoptosis [4]. Despite the fact that combination therapy might as well be toxic if one of the agents used is chemotherapeutic, the toxicity is lesser because different pathways would be targeted [4]. Moreover with the use of combination therapy, the toxicity on normal cells can be prevented while concurrently producing cytotoxic effects on cancer cells [4, 5].

In the recent past, virotherapy, a less toxic treatment has been identified as a possible cancer remedy [6–11]. Virotherapy involves the use of oncolytic viruses that infect, multiply, and directly lyse cancer cells with less or no toxicity [9]. Their tumor specific properties allow for viral binding, entry, and replication [12]. Oncolytic viruses can greatly enhance the cytotoxic mechanisms of chemotherapeutic drugs [13]. Further, chemotherapeutic drugs lyse fast multiplying cells and, in general, virus infected tumor cells quickly replicate [14].

Chemovirotherapy is a combination treatment strategy that involves the use of oncolytic viruses and chemotherapeutic drugs. Recent experimental and mathematical studies have shown that chemovirotherapy is a plausible cancer treatment and leads to enhanced therapeutic effects not achievable when either therapies are independently used [12, 13, 15–20]. Nguyen et al. [12] gave an account of the mechanisms through which drugs can successfully be used in a combination with oncolytic viruses. They however note that the success of this combination depends on several factors including the type of oncolytic virus- (OV-) drug combination used, the timing, frequency, dosage, and cancer type targeted. To date, the best method of OV drug delivery is debatable [21, 22].

The main goal of this study is to, thus, consider and compare the efficacy of three drug infusion methods, use mathematical analysis to predict the outcome of OV-drugs combination treatment and determine the effect of drug response and virus infection delays. To this end, we construct a mathematical model in the form of ODEs which we later extend to DDEs to include the virus infection and drug response delays. The model constructed combines elements from existing mathematical models [10, 11, 19, 20, 23–30]. Tian [10] presented a mathematical model that incorporates burst size for oncolytic virotherapy. His study showed that virotherapy is highly effective provided that viruses with large burst sizes are used. Malinzi et al. [19] constructed a spatiotemporal mathematical model to investigate the outcome of chemovirotherapy. Their study suggested that combining chemotherapeutic drugs with oncolytic viruses is more efficient than using either treatments alone. A similar study by Malinzi et al. [20] indicates that chemotherapy alone is capable of clearing tumor cells from body tissue if the drug efficacy is greater than the tumor growth rate. Nevertheless, the study contends that oncolytic viruses highly enhance chemotherapy in lysing tumor cells. The study further postulates that half the maximum tolerated doses of chemotherapy and virotherapy optimize chemovirotherapy, thus answering a very pertinent question in combination cancer therapy.

The article is organised as follows: Section 2 presents a comprehensive description of the both the ODE and DDE models and the underlying assumptions made in constructing them. In Section 3, the model without delay is analysed. First, without any form of treatment, then with either treatments (that is, with chemo only and virotherapy alone) and with both treatments. The delay model is then analysed in Section 4 and numerical experiments for both the ODE and DDE models are carried out in Section 5. Finally, before concluding in Section 7, a comparison of this study with related works is done is Section 6.

## 2. Model Description

### 2.1. Model without Delay

Time-dependent cell concentrations of uninfected tumor cells *U*(*t*), infected tumor cells *I*(*t*), a free virus population *V*(*t*), and a chemotherapeutic drug *C*(*t*) in an avascular tumor localization are considered. The uninfected tumor grows logistically at an intrinsic rate *α* per day, and the total tumor carrying capacity is *K* cells in a tumor nodule. The infected tumor cells die off at a rate *δ* per day. Virus multiplication in the tumor is represented by the function *βU*(*t*)*V*(*t*), where *β* is the virus replication rate measured per day per 10^6^ cells or viruses. The response of the drug to the uninfected and infected tumor is, respectively, modelled by the functions *δ*_0_ *U*(*t*)*C*(*t*) and *δ*_1_ *I*(*t*)*C*(*t*) where *δ*_0_ and *δ*_1_ are induced lysis rates caused by the chemotherapeutic drug measured per day per cell. Virus lifespan is taken to be 1/*γ* and its production is considered to be *bδI* where *b* is the virus burst size, measured in number of viruses per day per cell, and *δ* is the infected tumor cells' death rate measured per day. Chemotherapeutic drug infusion into the body is modelled with a function *g*(*t*) and that the drug gets depleted from body tissue at a rate *λ* per day.

Drug infusion into the body is simulated using (a) a constant rate *g*(*t*)=*q*, (b) an exponential *g*(*t*)=*q*  exp(−*at*), and (c) a sinusoidal function *g*(*t*)=*q*  sin^2^(*at*), where *q* is the rate of drug infusion. The constant *a* determines the exponential drug decay and period for the sinusoidal infusion. Constant drug infusion may relate to a situation where a patient is put on an intravenous injection or a protracted venous infusion and the drug is constantly pumped into the body [31, 32]. The exponential drug infusion may relate a situation where a cancer patient is given a single bolus and the drug exponentially decays in the body tissue. This form of infusion is not common although it is now used for some drugs, for example, a single dose of carboplatin can be given to patients with testicular germ cell tumors and breast cancer ([33, 34]). The third scenario is possible when a cancer patient makes several visits to a health facility and is given injections or anticancer drugs periodically [35, 36].

The assumptions above lead to the following system of nonlinear first-order differential equations (also similarly derived in [11, 19, 20]):

(1) $$\begin{array}{c} \dot{U}\left(t\right)=\alpha U\left(t\right)\left(1-\frac{U\left(t\right)+I\left(t\right)}{K}\right)-\beta U\left(t\right)V\left(t\right)-\delta_{0}U\left(t\right)C\left(t\right),\\ \dot{I}\left(t\right)=\beta U\left(t\right)V\left(t\right)-\delta I\left(t\right)-\delta_{1}I\left(t\right)C\left(t\right),\\ \dot{V}\left(t\right)=b\delta I\left(t\right)-\beta U\left(t\right)V\left(t\right)-\gamma V\left(t\right),\\ \dot{C}\left(t\right)=g\left(t\right)-\lambda C\left(t\right), \end{array}$$

subject to initial concentrations

(2) $$\begin{array}{c} U\left(0\right)=U_{0},\\ I\left(0\right)=I_{0},\\ V\left(0\right)=V_{0},\\ C\left(0\right)=C_{0}. \end{array}$$

### 2.2. Delay Model

The model is further extended to account for delays as a result of the infection of tumor cells by the virus and responses of the chemotherapeutic drug. In fact, the viruses need time to develop suitable responses when they meet the uninfected tumor cells (e.g., [37]). The drug does not instantaneously kill the cells (e.g., [38, 39]). By denoting the virus and chemotherapeutic response delays as *τ*_1_ and *τ*_2_, respectively, model (1)

(3) $$\begin{array}{c} \dot{U}\left(t\right)=\alpha U\left(t\right)\left(1-\frac{U\left(t\right)+\left(t\right)}{K}\right)-\beta U\left(t-\tau_{1}\right)V\left(t-\tau_{1}\right)-\delta_{0}U\left(t-\tau_{2}\right)C\left(t-\tau_{2}\right),\\ \dot{I}\left(t\right)=\beta U\left(t-\tau_{1}\right)V\left(t-\tau_{1}\right)-I\left(t\right)-\delta_{1}I\left(t-\tau_{2}\right)C\left(t-\tau_{2}\right),\\ \dot{V}\left(t\right)=bI\left(t\right)-\beta U\left(t-\tau_{1}\right)V\left(t-\tau_{1}\right)-\gamma V\left(t\right),\\ \dot{C}\left(t\right)=g\left(t\right)-\lambda C\left(t\right). \end{array}$$

## 3. Mathematical Analysis of Model without Delay

In this section, the model without delay (1) is analysed. The variables in system (1) are first rescaled by setting $\bar{t}=\delta t$, $U=K\bar{U}$, $I=K\bar{I}$, $V=V_{0}\bar{V}$, and $C=C_{0}\bar{C}$. Taking *V*_0_=*K*, the parameters are renamed to become

(4) $$\begin{array}{c} \bar{\alpha }=\frac{\alpha }{\delta },\bar{\beta }=\frac{\beta V_{0}}{\delta },\bar{\delta_{0}}=\frac{\delta_{0}C_{0}}{\delta },\bar{\delta_{1}}=\frac{\delta_{1}C_{0}}{\delta },\bar{b}=\frac{bK}{V_{0}},\bar{\gamma }=\frac{\gamma }{\delta },\phi =\frac{q}{\delta C_{0}},\psi =\frac{\lambda }{\delta },\bar{a}=\frac{a}{\delta }. \end{array}$$

For simplicity, we drop the bars and equation (1) becomes

(5) $$\begin{array}{c} \dot{U}\left(t\right)=\alpha U\left(t\right)\left(1-U\left(t\right)-I\left(t\right)\right)-\beta U\left(t\right)V\left(t\right)-\delta_{0}U\left(t\right)C\left(t\right),\\ \dot{I}\left(t\right)=\beta U\left(t\right)V\left(t\right)-I\left(t\right)-\delta_{1}I\left(t\right)C\left(t\right),\\ \dot{V}\left(t\right)=bI\left(t\right)-\beta U\left(t\right)V\left(t\right)-\gamma V\left(t\right),\\ \dot{C}\left(t\right)=\xi \left(t\right)-\psi C\left(t\right). \end{array}$$

*ξ*(*t*)=*ϕ*, *ξ*(*t*)=*ϕ*  exp(−*at*), and *ϕ*  sin^2^(*at*), respectively, are the constant, exponential, and sinusoidal infusion functions. For this model to be biologically meaningful, its solutions should be positive and bounded because they represent concentrations. Well-posedness theorems of model (5) are stated and proved in Appendix A.

### 3.1. Model Solutions

To investigate the efficacy of each treatment and their combination, we first study the dynamics of the system without treatment. Without any form of treatment, model (5) is reduced to only one equation:

(6) $$\begin{array}{c} \dot{U}\left(t\right)=\alpha U\left(t\right)\left(1-U\left(t\right)\right),\\ U\left(0\right)=U_{0}, \end{array}$$

whose solution is

(7) $$\begin{array}{c} U\left(t\right)=\frac{U_{0}}{\left(1-U_{0}\right)\exp\left(-\alpha t\right)+U_{0}},\\ \underset{t⟶\infty }{\lim}U\left(t\right)=1, \end{array}$$

implying that the tumor logistically grows to its maximum fractional size. Next, the model (5) is analysed, with chemotherapy, with virotherapy, and then with both treatments incorporated. We obtain, where possible analytical and time invariant solutions which predict the long term dynamics of the model equation (1).

Without virotherapy (*V*(*t*)=0), the system (5) is transformed to

(8) $$\begin{array}{c} \dot{U}\left(t\right)=\alpha U\left(t\right)\left(1-U\left(t\right)\right)-\delta_{0}U\left(t\right)C\left(t\right),\\ \dot{C}\left(t\right)=\xi \left(t\right)-\psi C\left(t\right), \end{array}$$

with *U*(0)=*U*_0_ and *C*(0)=*C*_0_. The second equation in equation (8) is a first-order linear ordinary differential equation which can easily be solved to give

(9) $$\begin{array}{c} C\left(t\right)=\exp\left(-\psi t\right)\left(\int \xi \left(t\right)\exp\left(\psi t\right)dt+R\right), \end{array}$$

where *R* is a constant of integration. The solution to the first equation in equation (8) depends on the infusion function *ξ*(*t*). For a fixed infusion function *ϕ*,

(10) $$\begin{array}{c} U\left(t\right)=\frac{e^{\left(\alpha t-\left(\delta_{0}\phi t/\psi \right)+\left(\delta_{0}e^{\left(-\psi t\right)}/\psi \right)\right)}}{\alpha \int e^{\left(\alpha t-\left(\delta_{0}\phi t/\psi \right)+\left(\delta_{0}e^{\left(-\psi t\right)}/\psi \right)\right)}dt+\left(e^{\delta_{0}\psi }/U_{0}\right)},\\ C\left(t\right)=\left(C_{0}-\frac{\phi }{\psi }\right)e^{-\psi t}+\frac{\phi }{\psi }. \end{array}$$

From the solution of *C*(*t*) in equation (10),

(11) $$\begin{array}{c} \underset{t⟶\infty }{\lim}C\left(t\right)=\frac{\phi }{\psi }. \end{array}$$

Biologically, it can be inferred that with a constant drug infusion and without virotherapy, the tumor is not completely cleared and a certain proportion of the drug remains in body tissue. The tumor clearance depends on the drug induced lysis of the tumor and the drug infusion rate which should be maximized and the tumor growth and drug decay rate which should be minimized.

For *ξ*(*t*)=*ϕ*  exp(−*at*),

(12) $$\begin{array}{c} U\left(t\right)=\frac{e^{\left(\left(a\alpha t/a-\psi \right)-\left(\alpha \psi t/a-\psi \right)+\left(c\delta_{0}e^{\left(-\psi t\right)}/a-\psi \right)-\left(ac\delta_{0}e^{\left(-\psi t\right)}/\left(a-\psi \right)\psi \right)+\left(\delta_{0}\phi e^{\left(-at\right)}/\left(a-\psi \right)a\right)\right)}}{a\int e^{\left(a\alpha t/a-\psi \right)-\left(\alpha \psi t/a-\psi \right)+\left(c\delta_{0}e^{\left(-\psi t\right)}/a-\psi \right)-\left(ac\delta_{0}e^{\left(-\psi t\right)}/\left(a-\psi \right)\psi \right)+\left(\delta_{0}\phi e^{\left(-at\right)}/\left(a-\psi \right)a\right)}dt+\left(e^{\left(c\delta_{0}/a-\psi \right)}/U_{0}\right)},\\ C\left(t\right)=\left(\frac{C_{0}\psi -\phi }{\psi }-\frac{\phi e^{\left(-at+\psi t\right)}}{a-\psi }\right)e^{\left(-\psi t\right)}, \end{array}$$

where *c*=(*C*_0_ *ψ* − *ϕ*/*ψ*).

From equation (12),

(13) $$\begin{array}{c} \underset{t⟶\infty }{\lim}C\left(t\right)=0,\\ \underset{t⟶\infty }{\lim}U\left(t\right)=U_{*}, \end{array}$$

where *U*_*∗*_ is a fractional tumor cell concentration between 0 and 1. This suggests that with a single dosage infusion of the chemotherapeutic drug with exponential decay and without virotherapy, the tumor cannot be cleared from body tissue. The drug is also completely depleted from the body.

When *ξ*(*t*)=*ϕ*  sin^2^(*at*) is substituted in equation (8), the resulting differential equations are solved to give

(14) $$\begin{array}{c} C\left(t\right)=\frac{1}{2}\left(2R-\frac{\left(\psi^{2}\cos\left(2at\right)e^{\left(\psi t\right)}+2a\psi e^{\left(\psi t\right)}\sin\left(2at\right)-\left(4a^{2}+\psi^{2}\right)e^{\left(\psi t\right)}\right)\phi }{4a^{2}\psi +\psi^{3}}\right)e^{\left(-\psi t\right)}, \end{array}$$

where

(15) $$\begin{array}{c} R=C_{0}+\frac{\psi^{2}-\phi \left(4a^{2}+\psi^{2}\right)}{2\psi \left(4a^{2}+\psi^{2}\right)},\\ \underset{t⟶\infty }{\lim}C\left(t\right)=C_{*}=f\left(a,\phi ,\psi \right). \end{array}$$

This suggests that with time, some drug concentration remains in the body tissue.

**Theorem 1 .**

The system (8), with constant infusion, has no periodic solutions for positive *U*(*t*) and *C*(*t*).

**Proof**

Using Dulac's criterion ([40]):

Suppose $\dot{X}=f\left(x\right)$ and *f*(*x*) is continuously differentiable on a simply connected domain *𝔻* ⊂ *ℝ*. If there exists a real valued function *g*(*x*) such that $\nabla \cdot \left(g\left(\dot{X}\right)\right)=\nabla \cdot \left(gf\right)$ has one sign in *𝔻*, then there are no closed orbits in *𝔻*. Using Dulac's criterion, it is sufficient to show that

(16) $$\begin{array}{c} \frac{\partial }{\partial U}\left(g\dot{U}\right)+\frac{\partial }{\partial C}\left(g\dot{C}\right)\neq 0,\;\forall U,C\in R_{+}^{2}. \end{array}$$

Consider

(17) $$\begin{array}{c} g\left(U,C\right)=\frac{1}{UC},\\ \nabla \cdot \left(g\dot{X}\right)=\frac{\partial }{\partial U}\left(g\dot{U}\right)+\frac{\partial }{\partial C}\left(g\dot{C}\right),\\ =-\left(\frac{\alpha }{C}+\frac{\xi \left(t\right)}{UC^{2}}\right)<0,\;\forall U,C\in {\left(R^{+}\right)}^{2}. \end{array}$$

**Theorem 2 .**

The system (8) has at least two steady states for each of the drug infusion functions:

1. For the constant drug infusion function *ξ*(*t*)=*ϕ*, there are two steady states of equation (8): (*U*=0, *C*=(*ϕ*/*ψ*)) which is locally asymptotically stable provided that *δ*_0_ *ϕ* > *αψ* and (*U*=1 − (*δ*_0_ *ϕ*/*αψ*), *C*=0) which is locally asymptotically stable provided that *δ*_0_ *ϕ*+*αψ* > 2*δ*_0_ *ϕψ* ^2^; otherwise, it is unstable.
2. For the exponential drug infusion *ξ*(*t*)=*ϕ*  exp(−*at*), equation (8) has two steady states: (*U*=0, *C*=0, *W*=0) which is unstable and (*U*=1, *C*=0, *W*=0) which is locally asymptotically stable.
3. For the sinusoidal infusion function, there are four steady states of equation (8): (*U*=0, *C*=0, *W*=0), (*U*=1, *C*=1, *W*=0), and (*U*=(*aαψ* − *δ*_0_ *ϕ*/*aαψ*), *C*=(*ϕ*/*aψ*), *W*=(*ϕ*/*a*)) which are unstable and (*U*=0, *C*=(*ϕ*/*aψ*), *W*=(*ϕ*/*a*)) which is locally asymptotically stable if *δ*_0_ *ϕ* > *aαψ* and *ϕ* < 1.

**Proof**

(1) It is easy to show that when equation (8) is equated to zero, one obtains two steady states. The characteristic polynomial of the Jacobian matrix for equation (8) evaluated at (0, (*ϕ*/*ψ*)) is
  (18) $$\begin{array}{c} \lambda^{2}+\left(-\alpha +\frac{\delta_{0}\phi }{\psi }+\psi \right)\lambda +\delta_{0}\phi -\alpha \psi , \end{array}$$
  whose roots *λ* can only be negative if *δ*_0_ *ϕ* > *αψ*.
The characteristic polynomial evaluated at (1 − (*δ*_0_ *ϕ*/*αψ*), 0) is
  (19) $$\begin{array}{c} \lambda^{2}+\left(-2\delta_{0}\phi \psi +\alpha +\frac{\delta_{0}\phi }{\psi }+\psi \right)\lambda -2\;\;\delta_{0}\phi \psi^{2}+\delta_{0}\phi +\alpha \psi , \end{array}$$
  whose roots are negative provided that *δ*_0_ *ϕ*+*αψ* > 2*δ*_0_ *ϕψ* ^2^.
(2) By letting *W*=*ϕ*  exp(−*at*), equation (8) is turned into an autonomous system:
  (20) $$\begin{array}{c} \dot{U}\left(t\right)=\alpha U\left(t\right)\left(1-U\left(t\right)\right)-\delta_{0}U\left(t\right)C\left(t\right),\\ \dot{C}\left(t\right)=W-\psi C\left(t\right),\\ \dot{W}\left(t\right)=-aW. \end{array}$$
The eigenvalues of the Jacobian matrix for equation (20) evaluated at (0,0,0) which are −*ψ*, *α*, and 0 and at (1,0,0) which are −*α*, −*ψ*, and −*a* are all negative.
(3) Similarly, by letting *W*=*ϕ*  sin^2^  *t*, equation (8) becomes the autonomous system:
  (21) $$\begin{array}{c} \dot{U}\left(t\right)=\alpha U\left(t\right)\left(1-U\left(t\right)\right)-\delta_{0}U\left(t\right)C\left(t\right),\\ \dot{C}\left(t\right)=W-\psi C\left(t\right),\\ \dot{W}\left(t\right)={\left[4aW\left(\phi -W\right)\right]}^{1/2}. \end{array}$$

The eigenvalues of the Jacobian matrix for equation (20) evaluated at (0,0,0) are −*ψ*, *α*, and 0 and the eigenvalues evaluated at (1,0,0) are *ψ*, −*α*, and 0. For the third steady state to exist, *aαψ* ≥ *δ*_0_ *ϕ*, and the eigenvalues evaluated at this state are $\left(-\left(a\alpha \psi -\delta_{0}\phi /a\psi \right),-\left(4a\phi /\sqrt{4\phi \left(1-\phi \right)}\right),-\psi \right)$, implying that for it to be locally asymptotically stable, *δ*_0_ *ϕ* > *aαψ*; yet for this to happen, the steady state will not exist. The eigenvalues evaluated at (*U*=0, *C*=(*ϕ*/*aψ*), *W*=(*ϕ*/*a*)) are $\left(-\left(a\alpha \psi -\delta_{0}\phi /a\psi \right),-\left(4a\phi /\sqrt{4\phi \left(1-\phi \right)}\right),-\psi \right)$ implying that this steady state is locally asymptotically stable if *δ*_0_ *ϕ* > *aαψ* and *ϕ* < 1.

Theorems 1 and 2 show that there are no periodic solutions in the dynamics of equation (8) and with a constant drug infusion, the tumor can be eliminated from body tissue by chemotherapy provided that the combination of the chemotherapeutic drug-induced lysis of the tumor and the drug infusion is greater than the combination of the intrinsic tumor growth rate and the drug deactivation rate. The tumor can also be wiped out with a periodic drug infusion provided that the combination of the tumor-induced lysis by the drug and the dosage is greater than the intrinsic tumor growth rate and drug decay rate. With the exponential infusion method, the tumor is not removed from body tissue and may grow to its maximum size.

Without chemotherapy, equation (5) is reduced to

(22) $$\begin{array}{c} \dot{U}\left(t\right)=\alpha U\left(t\right)\left(1-U\left(t\right)-I\left(t\right)\right)-\beta U\left(t\right)V\left(t\right),\\ \dot{I}\left(t\right)=\beta U\left(t\right)V\left(t\right)-I\left(t\right),\\ \dot{V}\left(t\right)=bI\left(t\right)-\beta U\left(t\right)V\left(t\right)-\gamma V\left(t\right). \end{array}$$

The analytical solutions to system (22) are not easy to obtain. The derivatives of equation (22) are therefore equated to zero to obtain time invariant solutions and investigate their stability by linearizing equation (22) about the steady states.

**Theorem 3 .**

(1) If *β*+*γ* > *bβ*, the system (12) has two steady states: a tumor free cell state (0,0,0) which is unstable and an infection tumor free state (1,0,0) which is locally asymptotically stable.
(2) If *bβ* > *β*+*γ*, the system (22) has three steady states: the tumor free state (0,0,0) and the infected free state (1,0,0) which are unstable and a tumor dormant state:
  (23) $$\begin{array}{c} \left(U=\frac{\gamma }{\left(b-1\right)\beta },I=\frac{\alpha \gamma \left(\beta \left(b-1\right)-\gamma \right)}{\beta \left(\beta {\left(b-1\right)}^{2}+\gamma \left(b-1\right)\right)},V=\frac{\alpha \left(\beta \left(b-1\right)-\gamma \right)}{{\left(b-1\right)}^{2}\beta^{2}+\alpha \gamma }\right),\;b>1, \end{array}$$
  which is locally asymptotically stable if *a*_0_, *a*_1_, *a*_2_ > 0 and *a*_1_ *a*_2_ > *a*_0_ where *a*_*i*_ are coefficients of the characteristic equation.

**Proof**

(1) The characteristic equation evaluated at (0,0,0) is
  (24) $$\begin{array}{c} \lambda^{3}+\left(\gamma -\alpha +1\right)\lambda^{2}+\left(\gamma -\alpha \gamma -\alpha \right)\lambda -\alpha \gamma =0,\\ \left(\lambda -\alpha \right)\left(\lambda +1\right)\left(\lambda +\gamma \right)=0, \end{array}$$
  from which *λ*_1_=*α*, *λ*_2_=−1 and *λ*_3_=−*γ*, thus rendering it unstable.
The characteristic equation evaluated at (1,0,0) is
  (25) $$\begin{array}{c} \left(\lambda +\alpha \right)\left(\lambda^{2}+\lambda \left(1+\beta +\gamma \right)+\beta +\gamma -b\beta \right)=0, \end{array}$$
  from which *λ*_1_=−*α* and *λ* ^2^+*λ*(1+*β*+*γ*)+*β*+*γ* − *bβ*=0 which are all negative since *β*+*γ* > *bβ*.
(2) The characteristic polynomial evaluated at the tumor dormant state is *λ* ^3^+*a*_2_ *λ* ^2^+*a*_1_ *λ*+*a*_0_=0, where
  (26) $$\begin{array}{c} a_{2}=\left(2A\alpha +A\beta +C\beta -\alpha +\gamma +1\right),\\ a_{1}=\left(2A^{2}\alpha \beta -A\alpha \beta +C\alpha \beta -Ab\beta +2A\alpha \gamma +C\beta \gamma +2A\alpha +A\beta +C\beta -\alpha \gamma -\alpha +\gamma \right),\\ a_{0}=-2A^{2}\alpha b\beta +2A^{2}\alpha \beta +A\alpha b\beta +C\alpha \beta \gamma -A\alpha \beta +2A\alpha \gamma +C\beta \gamma -\alpha \gamma , \end{array}$$
  and *A*, *B*, *C* are the coordinates of the tumor dormant state. Using Routh–Hurwitz stability criterion, this state will only be locally asymptotically stable if *a*_0_, *a*_1_, *a*_2_ > 0 and *a*_1_ *a*_2_ > *a*_0_.

Since the infected tumor-free state is undesirable, the reverse of the condition *β*+*γ* > *bβ* is necessary for tumor eradication from body tissue. In other words, *bβ* > *β*+*γ*, that is, the product of the virus replication rate and their burst size should be greater than the sum of the burst size and virus replication rate. We also notice from equation (23) that

(27) $$\begin{array}{c} \underset{\beta ⟶\infty }{\lim}U=\underset{b⟶\infty }{\lim}U=0. \end{array}$$

It is therefore evident that high virus replication rate *β* and burst size *b* lead to lower tumor cell concentrations. The steady-state solutions of equation (23) involve many parameters, thereby giving rise to large expressions in the conditions for its stability. It is therefore a difficult undertaking to infer biological implications from these conditions. Nevertheless, it can be observed that virotherapy may only succeed in eliminating cancer from body tissue when the virus deactivation rate is very small or even zero and the virus replication rate very high.

Next, the model with both treatments is analysed. For a constant drug infusion rate *ϕ*, the system (5) has three steady states;

(i) Tumor-free steady state:
  (28) $$\begin{array}{c} \left(U=0,I=0,V=0,C=\frac{\phi }{\psi }\right). \end{array}$$

Here, the tumor and viruses are cleared from body tissue by the coupled treatment and a fraction of the chemotherapeutic drug remains in body tissue. The eigenvalues of the Jacobian matrix evaluated at this state are
  (29) $$\begin{array}{c} \lambda_{1}=-\frac{\delta_{0}\phi -\alpha \psi }{\psi },\\ \lambda_{2}=-\frac{\delta_{1}\phi +\psi }{\psi },\\ \lambda_{3}=-\gamma ,\\ \lambda_{4}=-\psi , \end{array}$$
  implying that this desirable state is locally asymptotically stable if *δ*_0_ *ϕ* > *αψ*. From this condition, in order to clear a tumor, the combination of the rate at which the drug kills the uninfected tumor cells and the drug infusion must be higher than the tumor growth rate and deactivation of the drug from body tissue.

(ii) Infected tumor-free state:
  (30) $$\begin{array}{c} \left(U=1-\frac{\delta_{0}\phi }{\alpha \psi },I=0,V=0,C=\frac{\phi }{\psi }\right). \end{array}$$
In this state, the whole tumor is not cleared as a fraction of uninfected tumor cells remain and all the infected ones are cleared by the treatment combination. Using the parameter values in Table 1, the eigenvalues of the Jacobian matrix evaluated at the infected tumor-free state are 0.403, 8.13, and 2.598 ± 2.418*i*, implying that the infected tumor-free state is unstable.
(iii) Tumor dormant state:
  (31) $$\begin{array}{c} \left(U=\frac{\left(\delta_{1}\phi +\psi \right)\gamma }{b\psi \left(b-1\right)-\delta_{1}\phi \beta },I=\frac{\Gamma \gamma }{\left(\delta_{1}\phi -\left(b-1\right)\psi \right)\beta },V=\Gamma ,C=\frac{\phi }{\psi }\right), \end{array}$$
  where
  (32) $$\begin{array}{c} \Gamma =\frac{\left(\beta \delta_{0}\delta_{1}\phi^{2}-b\beta \delta_{0}\phi \psi -\alpha \beta \delta_{1}\phi \psi -\alpha \delta_{1}\gamma \phi \psi +\alpha b\beta \psi^{2}+\beta \delta_{0}\phi \psi -\alpha \beta \psi^{2}-\alpha \gamma \psi^{2}\right)}{\left(b-1\right)\beta^{2}\psi^{2}+\alpha \beta \gamma \psi^{2}-\beta^{2}\delta_{1}\phi \psi }. \end{array}$$

It is a difficult undertaking to investigate the stability of this state without substituting parameter values because of the many terms involved. Using the parameter values in Table 1, the eigenvalues are −0.031, −0.025, −1.01, and −8.13, implying that this state is stable.

With the consideration of an exponential infusion function *ξ*(*t*)=*ϕ*  exp(−*at*), the equation (5) are first turned into an autonomous system of differential equations by letting *W*=*ϕ*  exp(−*at*). This system has three steady states:

(i) A tumor-free state where all cell concentrations diminish to zero:
  (33) $$\begin{array}{c} \left(U=0,I=0,V=0,C=0,W=0\right). \end{array}$$
This state is unstable because the eigenvalues −*γ*, −*ψ*, −*a*, −1, and *α* are not all negative.
(ii) A state where the tumor grows to its maximum size:
  (34) $$\begin{array}{c} \left(U=1,I=0,V=0,C=0,W=0\right). \end{array}$$
(iii) The characteristic polynomial evaluated at this state is equation (25) and this state is locally asymptotically stable if *β*+*γ* > *βγ*, otherwise it is unstable.
  (35) $$\begin{array}{c} \left(U=\frac{\gamma }{\left(b-1\right)\beta },I=\frac{\left(\alpha b-\alpha \right)\beta \gamma -\alpha \gamma^{2}}{{\left(b-1\right)}^{2}\beta^{2}+\left(\alpha b-\alpha \right)\beta \gamma },V=\frac{\left(\alpha b-\alpha \right)\beta -\alpha \gamma }{\left(b-1\right)\beta^{2}+\alpha \beta \gamma },C=0,W=0\right),\;b>1. \end{array}$$

The eigenvalues evaluated at this steady state are also big expressions and difficult to analyse analytically and extract conditions for stability. With the set of parameter values in Table 1, the eigenvalues are −0.1, −8.13, 1.054, and −0.014 ± 0.085*i*, implying that this state is stable.

Similarly, with *ξ*(*t*)=*ϕ*  sin^2^(*at*), one changes equation (5) into an autonomous system of equations by letting *W*=*ϕ*  sin^2^(*at*). The autonomous system has six steady states:

(i) Tumor-free state where all cell concentrations are wiped out of body tissue:
  (36) $$\begin{array}{c} \left(U=0,I=0,V=0,C=0,W=0\right). \end{array}$$
The eigenvalues evaluated at this state are −*γ*, −*ψ*, −*a*, −1, and *α* implying that it is unstable.
(ii) A state where the tumor grows to its maximum size:
  (37) $$\begin{array}{c} \left(U=1,I=0,V=0,C=1,W=0\right). \end{array}$$
The condition for stability of this state is same as with the exponential drug infusion case, that is, the state is locally asymptotically stable if *β*+*γ* > *βγ*.
(iii) Tumor-free state where some concentration of the drug remains:
  (38) $$\begin{array}{c} \left(U=0,I=0,V=0,C=\frac{\phi }{a\psi },W=\frac{\phi }{a}\right). \end{array}$$
This state is locally asymptotically stable if *δ*_0_ *ϕ* > *αψ* and (1/2) < *ϕ* < 1 because the eigenvalues evaluated at this state are
  (39) $$\begin{array}{c} \frac{\delta_{0}\phi -\alpha \psi }{\psi },\\ -\frac{\delta_{1}\phi +\psi }{\psi },\\ -\frac{2\left(2a\phi \left(\phi -\phi \right)\right)}{\sqrt{4\phi \left(1-\phi \right)}},\\ -\gamma ,\\ -\psi , \end{array}$$
  otherwise, it is unstable.
(iv) Infected tumor-free state where all infected tumor cells are wiped but a certain proportion of the uninfected remains:
  (40) $$\begin{array}{c} \left(U=\frac{a\alpha \psi -\delta_{0}\phi }{a\alpha \psi },I=0,V=0,C=\frac{\phi }{a\psi },W=\frac{\phi }{a}\right),\;a\alpha \psi \geq \delta_{0}\phi . \end{array}$$
(v) Drug-free state where the chemotherapeutic drug is wiped out of body tissue and proportions of all the other cell concentrations remain:
  (41) $$\begin{array}{c} \left(U=\frac{\gamma }{\left(b-1\right)\beta },I=\frac{\left(\alpha b-\alpha \right)\beta \gamma -\alpha \gamma^{2}}{{\left(b-1\right)}^{2}\beta^{2}+\left(\alpha b-\alpha \right)\beta \gamma },V=\frac{\left(\alpha b-\alpha \right)\beta -\alpha \gamma }{\left(b-1\right)\beta^{2}+\alpha \beta \gamma },C=0,W=0\right),\;b>1. \end{array}$$
(vi) Tumor dormant state:
  (42) $$\begin{array}{c} U=\frac{\left(\delta_{1}\phi +a\psi \right)\gamma }{\left(ab-a\right)\beta \psi -\beta \delta_{1}\phi },I=\frac{\Gamma_{2}\gamma }{ab\psi -\delta_{1}\phi -a\psi },V=\frac{\gamma_{2}}{a\psi },C=\frac{\phi }{a\psi },W=\frac{\phi }{a}, \end{array}$$
  where
  (43) $$\begin{array}{c} \Gamma_{2}=\frac{a^{2}\alpha b\beta \psi^{2}-ab\beta \delta_{0}\phi \psi -a\alpha \beta \delta_{1}\phi \psi -a\alpha \delta_{1}\gamma \phi \psi -a^{2}\alpha \beta \psi^{2}-a^{2}\alpha \gamma \psi^{2}+\beta \delta_{0}\delta_{1}\phi^{2}+a\beta \delta_{0}\phi \psi }{\left(ab\beta \psi +a\alpha \gamma \psi -\beta \delta_{1}\phi -a\beta \psi \right)a\beta \psi }. \end{array}$$

The conditions for stability for the last three states all depend on huge expressions from which it is hard to extract meaningful biological implications. This analysis, however, suggests that with both treatments and using a sinusoidal type infusion, the tumor can be eliminated from body tissue provided that the combination of the drug infusion rate and the lysis rate of the tumor is greater than the combination of the tumor growth rate and rate of drug loss.

## 4. Mathematical Analysis of Delay Model

The nondimensionalised delay model is

(44) $$\begin{array}{c} \dot{U}\left(t\right)=\alpha U\left(t\right)\left(1-\frac{U\left(t\right)+\left(t\right)}{K}\right)-\beta U\left(t-\tau_{1}\right)V\left(t-\tau_{1}\right)-\delta_{0}U\left(t-\tau_{2}\right)C\left(t-\tau_{2}\right),\\ \dot{I}\left(t\right)=\beta U\left(t-\tau_{1}\right)V\left(t-\tau_{1}\right)-I\left(t\right)-\delta_{1}I\left(t-\tau_{2}\right)C\left(t-\tau_{2}\right),\\ \dot{V}\left(t\right)=bI\left(t\right)-\beta U\left(t-\tau_{1}\right)V\left(t-\tau_{1}\right)-\gamma V\left(t\right),\\ \dot{C}\left(t\right)=\phi \left(t\right)-\psi C\left(t\right). \end{array}$$

Without virotherapy the model (44) in nondimensional form becomes

(45) $$\begin{array}{c} \dot{U}\left(t\right)=\alpha U\left(t\right)\left(1-U\left(t\right)\right)-\delta_{0}U\left(t-\tau_{2}\right)C\left(t-\tau_{2}\right),\\ \dot{C}\left(t\right)=\phi \left(t\right)-\psi C\left(t\right). \end{array}$$

The system (45) has two steady states: a tumor-free state *T*_0_=(0, (*ϕ*/*ψ*)) and a tumor dormant state (1 − (*δ*_0_ *ϕ*/*αψ*), 0), just as previously seen. Letting *Z*_1_(*t*)=*U*(*t*) − *U* ^*∗*^ and *Z*_4_(*t*)=*C*(*t*) − *C* ^*∗*^ where *U* ^*∗*^, *C* ^*∗*^ are steady states of equation (45), the linearized model about (*U* ^*∗*^, *C* ^*∗*^) of equation (45) is

(46) $$\begin{array}{c} \dot{Z_{1}}\left(t\right)=\alpha Z_{1}\left(t\right)-2\alpha U^{*}Z_{1}\left(t\right)-\delta_{0}U^{*}Z_{4}\left(t-\tau_{2}\right)-\delta_{0}C^{*}Z_{1}\left(t-\tau_{2}\right),\\ \dot{Z_{4}}\left(t\right)=\phi \left(t\right)-\psi Z_{4}\left(t\right). \end{array}$$

The characteristic equation of equation (46) evaluated at the tumor-free steady state is

(47) $$\begin{array}{c} f\left(\lambda \right)=\left(\lambda +\psi \right)\left(\lambda -\alpha +\frac{e^{-\lambda \tau_{2}}\delta_{0}\phi }{\psi }\right)=0. \end{array}$$

For *τ*_2_=0, we obtain the same characteristic polynomial as in the ODE case (Theorem 2). For *τ*_2_ ≠ 0, equation (47) is a transcendental equation and therefore has infinitely many roots and also makes it nontrivial to determine these roots. Nonetheless, the following is noticed:

**Lemma 1 .**

1. The tumor-free state *T*_0_ is stable if *τ*_2_=(*ψ*/*δ*_0_ *ϕ*) and *α*=(*δ*_0_ *ϕ*/*ψ*), i.e., Equation (47) has a negative root −*ψ* and a zero double root.
2. *T*_0_ is stable for a sufficiently small *τ*_2_, i.e., Equation (47) has negative real roots for 0 ≤ *τ*_2_ ≤ *τ*_2_0__.
3. Equation (47) has a pair of purely imaginary roots ±*iω* if *αψ* ≤ *δ*_0_ *ϕ* and the other root is −*ψ*. Therefore, *T*_0_ is unstable.

**Proof**

(1) If *τ*_2_=(*ψ*/*δ*_0_ *ϕ*) and *α*=(*δ*_0_ *ϕ*/*ψ*), then
  (48) $$\begin{array}{c} \frac{df}{d\lambda }=2\lambda -\alpha +\frac{\delta_{0}\phi }{\psi }e^{-\lambda \tau_{2}}-\lambda \tau_{2}\frac{\delta_{0}\phi e^{-\lambda \tau_{2}}}{\psi }+\psi -\tau_{2}\delta_{0}\phi e^{-\lambda \tau_{2}},\\ \frac{d^{2}f}{d\lambda^{2}}=2-2\frac{\tau_{2}\delta_{0}\phi }{\psi }e^{-\lambda \tau_{2}}+\tau_{2}^{2}\delta_{0}\phi \left(\frac{\lambda }{\psi }+1\right). \end{array}$$
Form equation (48),
  (49) $$\begin{array}{c} {\left.\frac{df}{d\lambda }\right|}_{\lambda =0}=0,\\ {\left.\frac{d^{2}f}{d\lambda^{2}}\right|}_{\lambda =0}=\frac{\psi }{\delta_{0}\phi }>0. \end{array}$$
Thus, equation (47) has a double zero root.
(2) If we denote *ρ*(*τ*_2_)+*iω*(*τ*_2_) as the root of the equation (47), the tumor-free state is stable if *ρ*(0) < 0. By continuity, if *τ*_2_ > 0 is sufficiently small, we still have *ρ*(*τ*_2_) < 0 and the tumor-free state is stable.
(3) If equation (47) has only purely imaginary roots, then the roots should be solutions to the equation
  (50) $$\begin{array}{c} \lambda -\alpha +\frac{e^{-\lambda \tau_{2}}\delta_{0}\phi }{\psi }=0. \end{array}$$

Assume that *λ*=*iω*, *ω* > 0 is the root of equation (50). Substituting *λ*=*iω* and separating real and imaginary parts, one gets

(51) $$\begin{array}{c} -\alpha +\frac{\delta_{0}\phi }{\psi }\cos\;\;\omega \tau_{2}=0,\\ \omega -\frac{\delta_{0}\phi }{\psi }\sin\;\;\omega \tau_{2}=0. \end{array}$$

Squaring both equations and adding gives

(52) $$\begin{array}{c} \omega^{2}+\alpha^{2}-\frac{\delta_{0}^{2}\phi^{2}}{\psi^{2}}=0, \end{array}$$

from which $\omega =\pm \sqrt{\left(\left(\delta_{0}\phi /\psi \right)+\alpha \right)\left(\left(\delta_{0}\phi /\psi \right)-\alpha \right)}$ if *αψ* > *δ*_0_ *ϕ* and the critical values of *τ*_2_ are

(53) $$\begin{array}{c} \tau_{2_{k}}=\frac{1}{\omega_{+}}\left(\arccos\frac{\alpha \psi }{\delta_{0}\phi }\right),\;k=0,1,2,\ldots . \end{array}$$

Without chemotherapy equation (44) becomes

(54) $$\begin{array}{c} \dot{U}\left(t\right)=\alpha U\left(t\right)\left(1-U\left(t\right)\right)-\beta U\left(t-\tau_{1}\right)V\left(t-\tau_{1}\right),\\ \dot{I}\left(t\right)=\beta U\left(t-\tau_{1}\right)V\left(t-\tau_{1}\right)-I\left(t\right),\\ \dot{V}\left(t\right)=bI\left(t\right)-\beta U\left(t-\tau_{1}\right)V\left(t-\tau_{1}\right)-\gamma v\left(t\right). \end{array}$$

By letting *Z*_1_(*t*)=*U*(*t*) − *U* ^*∗*^, *Z*_2_(*t*)=*I*(*t*) − *I* ^*∗*^ and *Z*_3_(*t*)=*V*(*t*) − *V* ^*∗*^ where *U* ^*∗*^, *I* ^*∗*^, and *V* ^*∗*^ are steady states of equation (54), the linearized model about (*U* ^*∗*^, *I* ^*∗*^, *C* ^*∗*^) of equation (54) is

(55) $$\begin{array}{c} \dot{Z_{1}}\left(t\right)=\alpha Z_{1}\left(t\right)-2\alpha U^{*}Z_{1}\left(t\right)+\alpha I^{*}Z_{1}\left(t\right)-\alpha U^{*}Z_{2}\left(t\right)+\beta V^{*}Z_{1}\left(t-\tau_{1}\right)+\beta U^{*}Z_{3}\left(t-\tau_{1}\right),\\ \dot{Z_{2}}\left(t\right)=-\beta V^{*}Z_{1}\left(t-\tau_{1}\right)+\beta U^{*}Z_{3}\left(t-\tau_{1}\right)-Z_{2}\left(t\right),\\ \dot{Z_{3}}\left(t\right)=bZ_{2}\left(t\right)+\beta V^{*}Z_{1}\left(t-\tau_{1}\right)+\beta U^{*}Z_{3}\left(t-\tau_{1}\right)-\gamma Z_{3}\left(t\right). \end{array}$$

The characteristic equation of equation (55) at (1,0,0) is

(56) $$\begin{array}{c} \left(\lambda +\alpha \right)\left[\left(\lambda +1\right)\left(\lambda +\gamma +\beta e^{-\lambda \tau_{1}}\right)-b\beta e^{-\lambda \tau_{1}}\right]=0. \end{array}$$

For *τ*_1_=0, we retrieve the same results as in Theorem 3. For *τ* ≠ 0, we have a transcendental equation to solve.

## 5. Numerical Simulations

### 5.1. Parameter Values

The parameter values used were obtained from fitted experimental data for untreated tumors and virotherapy in mice [41]. The tumor carrying capacity, *K*, is taken to be 10^6^ cells per unit volume. The number of viruses produced per day *b* is to be in the range 10 − 1000 [42]. Drug infusion and decay rates *q* and *λ* agree with cancer pharmokinetic studies [33, 34].

### 5.2. Simulation Results

Numerical simulations of the models (5) and (45) are presented, first with monotherapies followed with combination treatment. In all simulations, unless stated otherwise, initial concentrations are considered to be *U*_0_=1, *I*_0_=0, *V*_0_=0.1, and *C*_0_=0.1 with a high fractional untreated tumor cell count to necessitate clinical intervention. The equations were integrated using a Runge–Kutta fourth-order scheme and implemented in MATLAB. It is worth noting that the scale for the time and concentrations is, respectively, 1 unit ≈2 days and 1 unit =10^6^ number of cells.

**Figure 1:**
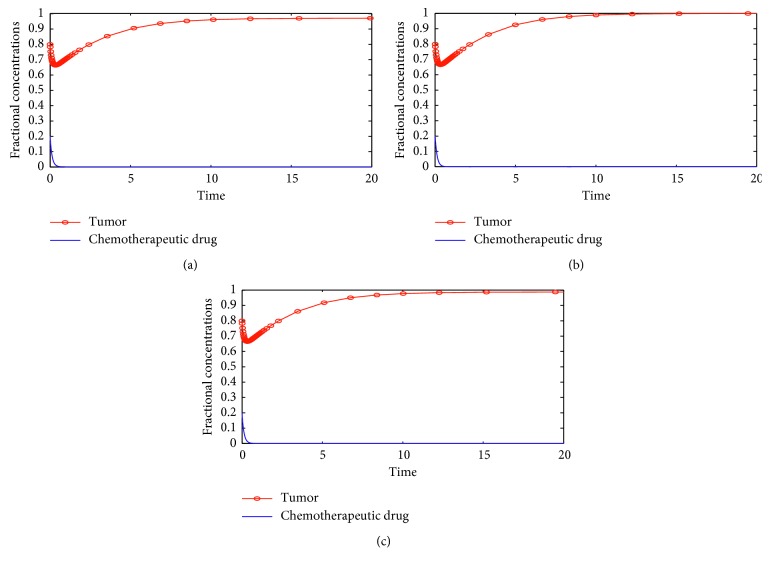
Solutions of the model without virotherapy: equation (5) showing a variation of fractional concentrations with time, using (a) a constant, (b) an exponential, and (c) a sinusoidal drug infusion. The initial cell concentrations are *U*_0_=0.8 and *C*_0_=0.2.

Figure 1 shows numerical solutions of the chemo-only model (8). The figure shows that despite the drug infusion method, the tumor is not cleared from body tissue. These numerical solutions agree with the analytical results obtained in the previous sections that chemotherapy on its own may not clear all tumor cells in body tissue, and the tumor grows to its maximum size and the drug concentration decays to zero. Section 3 revealed that total tumor clearance from body tissue can possibly be achieved if *δ*_0_ *ϕ* > *αψ*. Nonetheless, the parameter values used do not conform to this condition.

**Figure 2:**
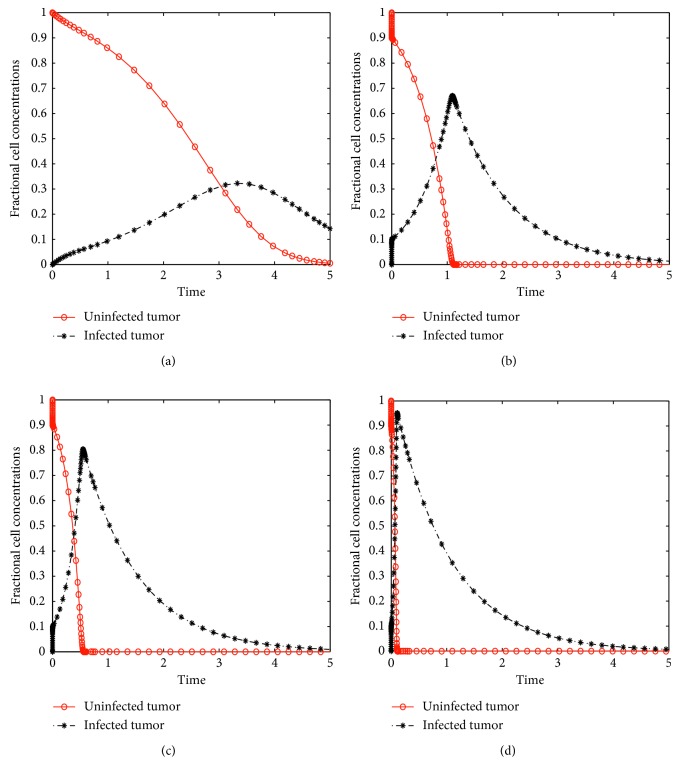
Solutions of model (12) without chemotherapy showing a variation of fractional concentrations with nondimensional time using low and high values of the virus replication rate *β* and burst size *b*, that is, (a) *β*=10^−6^, *b*=10; (b) *β*=10^−3^, *b*=10; (c) *b*=10, *β*=10^−6^; and (d) *b*=100, *β*=10^−6^.

Figure 2 shows the dynamics of the viro-only model. It is clear from this figure that virotherapy alone could possibly clear all tumor cells from body tissue provided that the virus infection rate is high and with a large virus burst size. Figures 2(a) and 2(b) show a variation of the fractional tumor and virus concentrations against time for different values of the virus replication rate. It is noticed that with a small virus infection rate for example *β*=10^−6^, it takes a longer time to clear the tumor cells. Figures 2(b) and 2(c) show a variation of the fractional concentrations with two different burst sizes. From these figures, we notice that when *b*=10, it takes about 10 days to reduce the whole tumor concentrations to zero while it takes only about 5 days with *b*=100, implying that a high virus burst size yields a quick recovery with virotherapy treatment. These numerical intimations concur with the analytical results established in Section 3.1.

**Figure 3:**
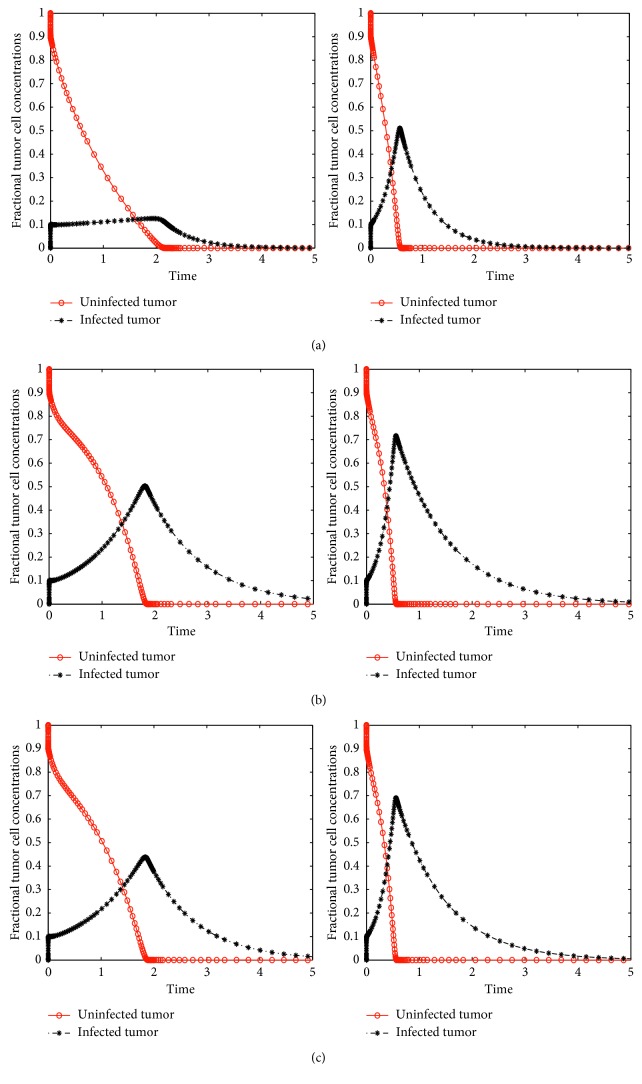
Solutions of model (3) with both treatments showing a variation of fractional concentrations with time using high and low virus burst sizes, that is, *b*=2 and *b*=5 and with different drug infusion functions, that is, (a) constant, (b) exponential, and (c) sinusoidal.

Figure 3 displays the dynamics of model (5) with both treatments. The numerical results are similar to those of Figure 2, only that with both treatments, it takes a shorter time to bring the tumor cell concentrations to zero. High values of the virus infection rate and burst size lead to tumor clearance in a shorter time period. Both Figures 2 and 3 show that an increase in the virus multiplication rate and burst size increase the infected tumor cells concentration. For example, comparing Figures 3(a) and 3(b), the number of infected tumor cells was about 0.15 × 10^6^ when *b*=15 and this increased to 0.35 × 10^6^ when *b*=25.

**Figure 4:**
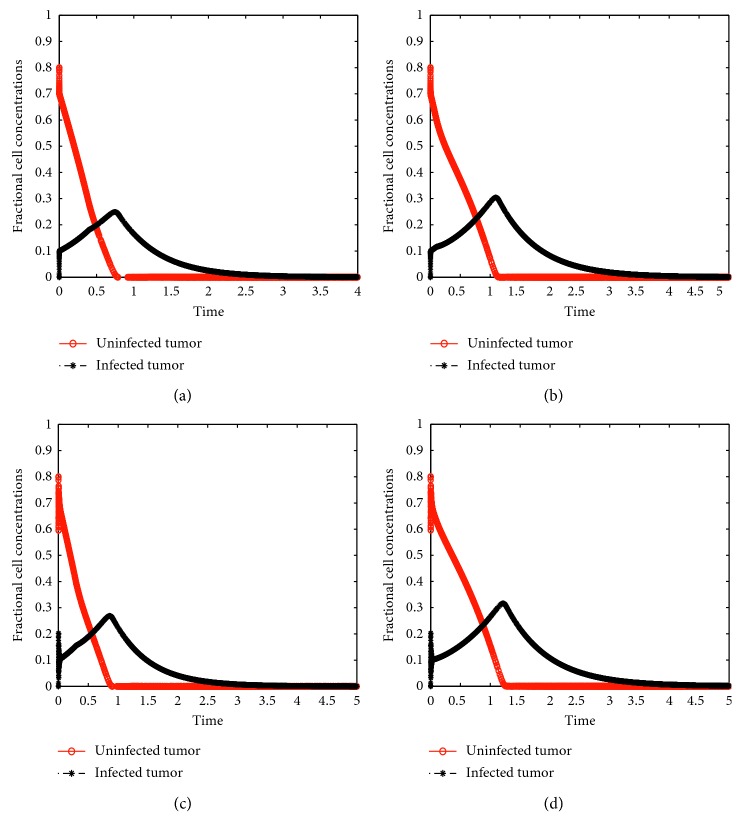
Solutions of delay model (15) showing a variation of fractional concentrations with time using (a) *τ*_1_=0.001, *τ*_2_=0.1; (b) *τ*_1_=0.01, *τ*_2_=0.1; (c) *τ*_1_=0.001, *τ*_2_=0.001; and (d) *τ*_1_=0.001, *τ*_2_=0.3.

**Figure 5:**
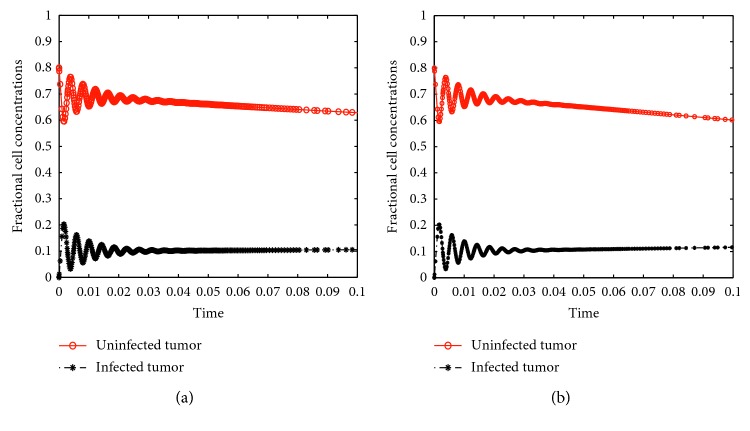
Close up images of Figures 4(c) and 4(d) showing oscillations in cell concentrations.

Model (44) was simulated using dde23 in MATLAB.  Figure 4 displays the numerical simulations for the delay model (44) with low and high values of *τ*_1_ and *τ*_2_, the virus, and chemotherapeutic delays. Since secondary transcription and viral protein synthesis can be delayed by about six to eight hours [37], *τ*_1_ was considered to be between 0.001 and 0.01. The chemotherapeutic response delay is higher than the virus response delay, thus *τ*_2_ was taken to be between 0.1 and 0.3. The figure shows that when both delays are increased, the time it takes for cell concentration solutions to converge is slightly increased although they converge at the same steady states as without the delay. For *τ*_1_=0.001 in Figure 4(a), it took about 4 days for the whole tumor to clear whereas it took about 8 days with *τ*_1_=0.01 in Figure 4(b). Comparing Figures 4(b) and 4(c), when *τ*_2_ was increased from 0.001 to 0.3, the time it took for the whole tumor to be cleared was increased from six to eight days. Initially there are oscillations for high values of the chemotherapeutic delay although the cell concentrations converge at the same steady states, just as the case with no delays.  Figure 5 is a close up form of Figure 4 to display oscillations caused by the virus and drug delays. The oscillations only occur in the initial stages of treatment but later fade away. Nonetheless, the results in Figures 4 and 5 suggest that it is imperative to design viruses and drugs which are highly responsive in order to minimize these delays.

## 6. Discussion

The results in this study contend with previous experimental and mathematical studies that oncolytic viruses enhance chemotherapeutic drugs in the treatment of cancer [12,13,15–20]. A study by Ungerechts et al. [16] examined the synergy between a reprogrammed oncolytic virus and two chemotherapeutic drugs in the mantle cell lymphoma (MCL). They investigated the efficacy of different procedures of a measles virus in combination with fludarabine and cyclophosphamide (CPA). Their study suggested that that CPA administration before virotherapy enhanced oncolytic efficacy. An experimental study by Ulasov et al. [17] indicated that the combination of virotherapy and temozolomide is capable of eliminating malignant glioma. Their results showed that 90% of treated mice survived beyond the 100 days' mark after being treated. Another study by Alonso et al. [18] showed that the amalgamation of oncolytic adenovirus (ICOVIR-5) with either everolimus (RAD001) or temozolomide (TMZ) resulted in an enhanced antiglioma effect. Recent mathematical studies by Malinzi et al. [19, 20] assert that combining chemotherapeutic drugs with oncolytic viruses is more efficient than using either treatments alone. In [20], it is indicated that although chemotherapy alone may clear tumor cells from body tissue if drug efficacy is bigger than the tumor growth rate, the use of both OV and drugs leads to enhanced treatment effects.

Biologically, the reduction of a tumor to undetectable levels in less than a week is unrealistic in comparison to existing clinical and research studies [43]. The duration of cancer treatment depends on several factors including the type of cancer being treated and the patient cells' characteristics. This makes it hard to predict the time period to clear a tumor in body tissue. Moreover, a tumor can be reduced to insignificant levels but may later regrow [44]. Nevertheless, this study agrees with the fact that chemovirotherapy is highly likely to bring the tumor to undetectable levels in a short time period just as previously established in [19, 20].

The mathematical model considered in this study is built on a couple of simplifying assumptions and thus omits pertinent biological aspects. For example, the drug infusion functions considered are not pragmatic. It is thus imperative to extend this study to consider more realistic drug infusion functions that describe all the important pharmacodynamics properties. Nonetheless, this study indicates that a cancer patient should not be given a single bolus injection as it is less effective compared to periodic or constant drug infusions.

## 7. Conclusion

The aim of this study was to investigate the outcome of the amalgamation of chemotherapy and virotherapy in treating cancer using three different drug infusion methods and to compare the efficacy of using chemotherapy and virotherapy individually.

A mathematical model in the form of nonlinear and nonautonomous first-order ordinary differential equations was developed. It was extended into delay differential equations to account for delays as a result of the infection of tumor cells by the virus and chemotherapeutic drug responses. The model's well-posedness was shown by proving existence, positivity, and boundedness of the model solutions. Analysis of the model was done with each of the treatments and for each of the infusion functions. Exact solutions were determined where possible. Stability of the time invariant solutions was carried out to determine the conditions under which a tumor-free situation may be achieved. Numerical simulations for the ODE and DDE models were, respectively, carried out using ode23s and dde23 in MATLAB. The model analysis suggested the following:

- A tumor can grow to its maximum size in case where there is no treatment.
- Chemotherapy alone is capable of clearing tumor cells in body tissue provided that the drug-induced lysis of the tumor and the drug infusion rate are maximized and the drug decay and tumor growth are minimized.
- Constant and periodic drug infusions are more potent than a single bolus injection.
- Successful virotherapy is highly dependent on virus burst size and infection rate.
- With the use of both chemotherapy and virotherapy, a tumor may be cleared from body tissue in less than a month.
- Successful chemovirotherapy depends on the virus burst size and replication rate, chemotherapeutic drug lysis, infusion and decay rates, and the method of drug infusion.
- Both the virus and chemotherapeutic response delays increase the period within which a tumor can be cleared from body tissue and thus treatment options should strive to minimize them by designing viruses and drugs which are highly responsive.

## Figures and Tables

**Table 1:** Dimensional parameter values.

*K*	*α*	*β*	*δ*	*γ*	*b*	*q*	*λ*	*δ*_0_	*δ*_1_
10^6^	0.206	0.001	0.5115	0.001	10	5	4.16	0.005	0.006
[41]	[41]	[41]	[41]	[41]	[42]	[23]	[23]	Estimated	Estimated

## Data Availability

The data used to support the findings of this study are available from the corresponding author upon request.

## Appendix

### A.1. Existence and Uniqueness

Theorem 4 . There exists a unique solution to the system of equation (5) in the region (*U*, *I*, *V*, *C*) ∈ *ℝ*_+_^4^.

Proof The Picard–Lindelöf theorem [45] is used as follows. Consider the closed interval *I*_*T*_=[*t*_0_ − *T*, *t*_0_+*T*] and the closed ball *B*_*d*_={*y* ∈ *ℝ* ^*n*^|‖*y* − *x*_0_‖ ≤ *d*} in *ℝ* ^*n*^ where *T* and *d* are positive, real numbers. Suppose that the function

(A.1) $$\begin{array}{c} f:I_{T}\times B_{d}⟶R^{n}, \end{array}$$

is continuous and that the partial derivatives in the Jacobian matrix *D*_*f*_ where

(A.2) $$\begin{array}{c} D_{\text{f}}=\left(\begin{array}{cccc} \frac{\partial f_{1}}{\partial x_{1}} & \frac{\partial f_{1}}{\partial x_{2}} & \cdots & \frac{\partial f_{1}}{\partial x_{n}}\\ \frac{\partial f_{2}}{\partial x_{1}} & \frac{\partial f_{2}}{\partial x_{2}} & \cdots & \frac{\partial f_{2}}{\partial x_{n}}\\ \vdots & \vdots & \ddots & \vdots \\ \frac{\partial f_{n}}{\partial x_{1}} & \frac{\partial f_{n}}{\partial x_{2}} & \cdots & \frac{\partial f_{n}}{\partial x_{n}} \end{array}\right), \end{array}$$

exist and are continuous in *I*_*T*_ × *B*_*d*_. Then, there exists a *δ* > 0 so that the initial value problem

(A.3) $$\begin{array}{c} \frac{dx}{dt}\left(t\right)=f\left(t,x\right),\\ x\left(t_{0}\right)=x_{0}, \end{array}$$

has a unique solution on the interval *I*_*δ*_=[*t*_0_ − *δ*, *t*_0_+*δ*]. It is sufficient to show that

(A.4) $$\begin{array}{c} f=\left(\begin{array}{c} f_{1}≔\alpha U\left(t\right)\left(1-U\left(t\right)-I\left(t\right)\right)-\beta U\left(t\right)V\left(t\right)-\delta_{0}U\left(t\right)C\left(t\right)\\ f_{2}≔\beta U\left(t\right)V\left(t\right)-\delta I\left(t\right)-\delta_{1}I\left(t\right)C\left(t\right)\\ f_{3}≔bI\left(t\right)-\beta U\left(t\right)V\left(t\right)-\gamma V\left(t\right)\\ f_{4}≔\xi \left(t\right)-\psi C\left(t\right) \end{array}\right), \end{array}$$

(A.5) $$\begin{array}{c} D_{\text{f}}=\left(\begin{array}{cccc} \frac{\partial f_{1}}{\partial U} & \frac{\partial f_{1}}{\partial I} & \frac{\partial f_{1}}{\partial V} & \frac{\partial f_{1}}{\partial C}\\ \frac{\partial f_{2}}{\partial U} & \frac{\partial f_{2}}{\partial I} & \frac{\partial f_{2}}{\partial V} & \frac{\partial f_{2}}{\partial C}\\ \frac{\partial f_{3}}{\partial U} & \frac{\partial f_{3}}{\partial I} & \frac{\partial f_{3}}{\partial V} & \frac{\partial f_{3}}{\partial C}\\ \frac{\partial f_{4}}{\partial U} & \frac{\partial f_{4}}{\partial I} & \frac{\partial f_{4}}{\partial V} & \frac{\partial f_{4}}{\partial C} \end{array}\right), \end{array}$$

exist and are continuous on *ℝ*_+_^4^. The functions (A.4) and (A.5) are polynomials and are therefore continuous on *ℝ* ^4^.

### A.2. Boundedness and Positive Invariance

Theorem 5 . if *U*(0) ≥ 0, *I*(0) ≥ 0, *V*(0) ≥ 0, and *C*(0) ≥ 0, then *U*(*t*) ≥ 0, *I*(*t*) ≥ 0, *V*(*t*) ≥ 0, and *C*(*t*) ≥ 0 for all *t* ≥ 0.

The same idea as in [10] is used to prove positiveness of the model solutions.

Proof Assuming that the Theory is not true, then there must be a time *t*_1_ such that at least one of the solutions becomes zero first. Each possible case is investigated; if *U*(*t*_1_)=0 first, then $\dot{U}\left(t_{1}\right)=0$. However, from the first equation in system (5), by the uniqueness of the solution, we know that *U*(*t*)=0 for all *t* ≥ *t*_1_. The second equation then becomes $\dot{I}\left(t\right)=-I\left(t\right)-\delta I\left(t\right)C\left(t\right)$ and its solution is

(A.6) $$\begin{array}{c} I\left(t\right)=I\left(t_{1}\right)\exp\left(-\int_{t_{1}}^{t}\left(1+\delta_{1}C\left(s\right)\right)ds\right),\;I\left(t\right)\geq 0. \end{array}$$

The third equation becomes $\dot{V}\left(t\right)=bI\left(t\right)-\gamma V\left(t\right)$. If you set

(A.7) $$\begin{array}{c} \dot{V}\left(t\right)=bI\left(t\right)-\gamma =0\geq -\gamma V\left(t\right)\;\;so\;\;V\left(t\right)\geq V\left(t_{1}\right)\exp\left(-\gamma t\right),\;V\left(t\right)\geq 0. \end{array}$$

Similarly, the fourth equation becomes $\dot{C}=\xi \left(t\right)-\psi C\left(t\right)$ and its solution is

(A.8) $$\begin{array}{c} C\left(t\right)=\exp\left(-\psi t\right)\left(\int_{t_{1}}^{t}\phi \left(s\right)\exp\left(\psi s\right)ds+C\left(t_{1}\right)\right), \end{array}$$

which implies that *C*(*t*) ≥ 0 for all *t* ≥ 0. If *I*(*t*_1_)=0 first, then $\dot{I}\left(t_{1}\right)=\beta U\left(t_{1}\right)V\left(t_{1}\right)\geq 0$, implying that when *t* > *t*_1_, *I*(*t*) ≥ 0 since *U*(*t*), *V*(*t*), *C*(*t*) ≥ 0 as assumed. If *V*(*t*_1_)=0 first, $\dot{V}\left(t_{1}\right)=by\left(t_{1}\right)\geq 0$, implying that when *t* ≥ *t*_1_, *V*(*t*) ≥ 0 since *U*(*t*), *I*(*t*), *C*(*t*) ≥ 0 as assumed. If *C*(*t*_1_)=0 first, $\dot{C}\left(t_{1}\right)=\phi \left(t_{1}\right)\geq 0$, so when *t* ≥ *t*_1_, *C*(*t*) ≥ 0 since *U*(*t*), *I*(*t*), *C*(*t*) ≥ 0 as assumed. If two solutions are zero (eg., *U*(*t*_1_)=0 and *I*(*t*_1_)=0) simultaneously at *t*=*t*_1_, then following the same steps above, it is trivial to check that the other solutions will be nonnegative for *t* > *t*_1_. If three solutions are zero (eg., *U*(*t*_1_)=0, *I*(*t*_1_)=0, and *V*(*t*_1_)=0) simultaneously at *t*=*t*_1_, it is trivial to check that the other solution will be nonnegative for *t* > *t*_1_. If the four solutions are zero simultaneously at *t*=*t*_1_, from the uniqueness theorem, *U*(*t*)=*I*(*t*)=*V*(*t*)=*C*(*t*)=0 for *t* > *t*_1_.

Theorem 6 . The trajectories evolve in an attracting region *𝔻*={(*U*, *I*, *V*, *C*) ∈ *ℝ*_+_^4^|*U*(*t*)+*I*(*t*) ≤ 1, *V*(*t*) ≤ (*b*/*γ*), *C*(*t*) ≤ *C*(*ϕ*)}, where *C*(*ϕ*) depends on the drug infusion function used.

Proof From equation (1), we know that *U*+*I* ≤ *K*. This implies that *U*+*I* ≤ 1.

(A.9) $$\begin{array}{c} \dot{V}\left(t\right)\leq bI\left(t\right)-\gamma V\left(t\right),\\ \dot{V}\left(t\right)\leq b-\gamma V\left(t\right),\\ V\left(t\right)\leq \frac{b}{\gamma }-\frac{V_{0}\exp\left(-\gamma t\right)}{\gamma },\\ \underset{t⟶\infty }{\lim}V\left(t\right)\leq \frac{b}{\gamma },\\ \dot{C}\left(t\right)+\psi C\left(t\right)=\xi \left(t\right),\\ C\left(t\right)=\exp\left(-\psi t\right)\left(\int \xi \left(t\right)\exp\left(\psi t\right)dt+R\right), \end{array}$$

where *R* is an arbitrary constant of integration. For the constant infusion function *ϕ*, lim_*t*⟶*∞*_ *C*(*t*)=*ϕ*/*ψ*. For *ξ*(*t*)=*ϕe* ^−*at*^, lim_*t*⟶*∞*_ *C*(*t*)=0, and for *ξ*(*t*)=*ϕ*  sin^2^(*at*), lim_*t*⟶*∞*_ *C*(*t*)=(*ψ*+2*a*/4*a* ^2^+*ψ* ^2^) − (*ϕ*/*ψ*).

Theorem 7 . The domain *𝔻* is positive invariant for the model equation (5) and therefore biologically meaningful for the tumor, virus, and drug cell concentrations.

Proof The proof directly follows from proofs of Theorems 5 and 6.
